# Fear conditioning and the basolateral amygdala

**DOI:** 10.12688/f1000research.21201.1

**Published:** 2020-01-28

**Authors:** Yajie Sun, Helen Gooch, Pankaj Sah

**Affiliations:** 1 Queensland Brain Institute, University of Queensland, Queensland, Australia; 2 Brain Research Centre and Department of Biology, Southern University of Science and Technology, Shenzhen, China

**Keywords:** learning, memory storage, associative learning, anxiety, long term potentiation

## Abstract

Fear is a response to impending threat that prepares a subject to make appropriate defensive responses, whether to freeze, fight, or flee to safety. The neural circuits that underpin how subjects learn about cues that signal threat, and make defensive responses, have been studied using Pavlovian fear conditioning in laboratory rodents as well as humans. These studies have established the amygdala as a key player in the circuits that process fear and led to a model where fear learning results from long-term potentiation of inputs that convey information about the conditioned stimulus to the amygdala. In this review, we describe the circuits in the basolateral amygdala that mediate fear learning and its expression as the conditioned response. We argue that while the evidence linking synaptic plasticity in the basolateral amygdala to fear learning is strong, there is still no mechanism that fully explains the changes that underpin fear conditioning.

## Introduction

Fear is a response to impending threat that prepares a subject to make appropriate defensive responses. Conserved across species, it describes a physiological state preparing the animal to freeze, fight, or flee to safety and, in humans, is accompanied by affective feelings of dread and anticipation. Our physiological understanding of fear and the neural circuits that underpin it have largely been studied using the Pavlovian paradigm of fear conditioning ^1^. In this paradigm, subjects, typically laboratory rodents, are exposed to a neutral sensory stimulus, such as a light, odor, or tone (the conditioned stimulus [CS]) that is contingently paired with an aversive one (the unconditioned stimulus [US]), typically a footshock. Following a number of pairings, sometimes just one, subjects exhibit defensive responses when exposed to the CS alone (the conditioned response [CR]). This learning is rapid and long lasting: presentation of the same CS days, weeks, or months after its conditioning continues to evoke defensive responses. Thus, fear conditioning is a form of associative learning in which pairing the CS with the US forms a memory trace that is later retrieved by the CS alone. As such, understanding the biology that underpins fear learning will not only help us understand fear but also provide insight into memory formation, storage, and retrieval. While there are differences between the subjective state of fear and anxiety ^2^, there are similarities in the accompanying physiological response, and the two states share neural circuits ^3– 5^. Thus, understanding the neural circuits that mediate fear may also help to unravel those that underpin anxiety disorders.

Like all learning, fear conditioning has three phases: acquisition, during which a sensory input; the CS, becomes associated with an aversive outcome; storage, in which a memory trace is formed; and retrieval, when the memory trace is retrieved and initiates defensive responses. Early lesion experiments established that the amygdala, a region in the mid-temporal lobe, is an essential component of the circuits that mediate fear learning ^6, 7^. The amygdala is a heterogeneous structure made of a number of nuclei that receive input from a host of cortical and subcortical areas and have extensive internuclear connectivity ^8^. Of these, the best understood are the basolateral amygdala (BLA) and central amygdala (CeA), which form the main input and output structures of the amygdala, respectively ^8– 10^. CS and US information converge in the BLA ^8– 11^, and contingent activation of these inputs forms a memory trace that may even be stored there ^12– 14^. Subsequent presentation of the CS activates circuits in the BLA, and projections from the BLA to the CeA drive defensive behaviors. While it is becoming increasingly clear that the CeA also plays a role in fear learning ^15, 16^, much work has gone into understanding the acquisition and processing of information in the BLA during fear learning and expression. In this brief review, we focus on the BLA and its role in a commonly studied form of associated learning: cued auditory fear conditioning.

## The basolateral amygdala

The BLA is located in the mid-temporal pole and anatomically divided into the lateral (LA) and basal (BA) nuclei. The LA is situated dorsal to the BA and is subdivided into the dorsolateral (LAdl), ventrolateral (LAvl), and ventromedial nuclei ^17^, while the BA consists of the basolateral nucleus (BL) and the basomedial nuclei (BM), also known as the accessory BA (AB) ^8, 18– 20^. These divisions within the BLA are cytoarchitectonically different and have different internuclear and extranuclear connections ^18, 19, 21^. For example, the BL is subdivided into the rostral magnocellular subdivision and the more caudal intermediate and parvicellular subdivisions, while the AB comprises the magnocellular subdivision and the more medial and caudal parvicellular subdivision ^8, 20^.

## Fear conditioning: acquisition

Associative fear learning has an absolute requirement for CS–US contingency—that is, a temporal relationship between the two stimuli, and learning is weakened when this contingency is broken ^1^. In auditory fear conditioning, the CS (tones) and US (footshock) inputs converge on to single neurons in the LA ^22– 24^. The prevailing model for associative learning is that conjunction of CS and US input results in long-term potentiation (LTP) ^25, 26^ of synapses carrying CS information, and this underpins the memory of the aversive nature of the CS ^9, 11, 27– 29^. CS and US inputs use glutamate as the excitatory transmitter, and these inputs form classical dual-component glutamatergic synapses that express postsynaptic AMPA and N-methyl-D-aspartate (NMDA) receptors ^30, 31^. NMDA receptors are calcium-permeable, cationic ion channels that are open only when the glutamate site on the receptor is occupied and the membrane potential is depolarised ^32, 33^. Thus, these receptors are coincidence detectors ^34^, and cytosolic calcium delivered by their activity is required for many forms of synaptic plasticity ^35, 36^. Fear conditioning requires NMDA receptor activity in the BLA ^37– 39^, and CS inputs are known to undergo plasticity following fear learning ^40, 41^. Thus, it is generally accepted that NMDA receptor-dependent LTP underpins Pavlovian fear conditioning ^11, 28, 42– 44^. In this model, the CS engages glutamatergic synapses, and the US provides the coincident depolarizing signal that drives NMDA receptor activity, triggering LTP of inputs carrying CS information ^11, 39, 43, 45^.

While this model is compatible with much of the literature and provides a plausible model for fear learning, how CS–US pairings result in LTP of synapses carrying CS input is not clear. In most auditory fear conditioning protocols, the CS lasts several seconds and then co-terminates with the US (known as delay fear conditioning). Typically, a 10-second CS is used, with the US being presented in the last 1 second and co-terminating with the CS. However, in LA principal neurons, the response to prolonged auditory stimulation is transient, lasting at most several hundred milliseconds ^22, 46, 47^. Whole cell recordings *in vivo* also show auditory evoked synaptic activity to last only a short period of time ^23^, suggesting that synapses carrying CS information are not active at the time the US signal arrives in the BLA. Moreover, when the CS and US are separated by a brief period of time, a procedure called trace conditioning, fear conditioning can still be induced ^48^. While trace conditioning with a long trace interval (>5 seconds) engages the hippocampus ^48, 49^, perhaps indicating a different form of learning, this does not happen with short (<3 seconds) trace intervals. In these experiments, although the interval between the CS and US is short, the offset time constant of synaptic NMDA receptors in the BLA is much shorter (in the order of ~100 milliseconds) ^50, 51^, meaning that with trace intervals of >1 second, ionotropic glutamate receptors mediating CS information are again not engaged when the US signal arrives. Thus, one requirement for NMDA receptor-dependent LTP, receptor engagement by glutamate, is not met. Furthermore, while neurons in the BLA receive both CS and US input, some neurons that change their response to the CS appear to not respond to the US ^52^, an observation that challenges the requirement of contingent input onto single neurons.

Finally, it is well established that the recent history of the CS is an important determinant in learning. One example is the blocking effect ^1^, in which a compound CS (light + tone) is paired with the US. If one of the CSs (e.g. the tone) has previously been paired with the US, subjects do not develop defensive responses to the light ^53, 54^. This result suggests that factors other than a close temporal relationship between the CS and US are required in fear associative learning. Interestingly, the US has been found to activate several ascending systems that release neuromodulators such as noradrenaline ^55^ and acetylcholine ^56^, and these systems are known to be involved in fear learning. However, how activation of these neuromodulatory systems modulates NMDA receptor-driven plasticity evoked during acquisition is not currently clear. In summary, in cued fear conditioning, it is clear that CS–US contingency is necessary for associative learning, and while the idea that synaptic plasticity (LTP) within the BLA underpins learning is very compelling, how this plasticity is evoked is still not clear.

## Fear conditioning: the role of inhibition

The BLA is a cortical-like structure, with the majority of neurons (principal or pyramidal) being glutamatergic and the rest (~20%) being GABAergic inhibitory interneurons ^57– 59^.

Although relatively a smaller population, interneurons powerfully regulate the excitability of principal cells ^15, 60– 63^. Thus, within the BLA, principal cells have very low resting firing rates ^64^ and single interneurons can block their activity ^63^. The importance of inhibition in fear learning was established early with experiments showing that pharmacologically enhancing inhibition in the BLA is anxiolytic and can block fear learning ^65, 66^, and *in vitro* studies show that plasticity of thalamic and cortical input to BLA principal cells is strongly modulated by inhibition ^60, 67, 68^.

Similar to the cortex, interneurons are divided into distinct families based on expression of cytosolic markers and synaptic connections ^15, 59, 69– 72^. Of these, the major population are interneurons that express calbindin and those that express calretinin ^73, 74^. These groups can be further subdivided based on their expression of neuropeptides such as somatostatin (SOM) or the calcium-binding protein parvalbumin (PV) ^15, 59, 69, 72^, with PV interneurons being more numerous in the BA as compared to the LA ^74^. Recent work has focused on these latter two families, which have distinct subcellular targets on principal neurons ^15, 59, 75^. PV interneurons innervate the somatic and proximal dendritic compartment, as well as the axon initial segment ^76– 79^, the likely site of action potential generation, while SOM interneurons target the distal dendritic tree. Both PV and SOM interneurons provide feedforward as well as feedback inhibition. In the LA, fast spiking interneurons (likely PV interneurons) have been found to receive cortical and thalamic inputs, again indicating a role in feedforward inhibition ^80– 82^. While the exact source of afferent inputs to these interneuron types has not been fully characterized, PV interneurons in the BA have been suggested to have both feedforward ^83^ and feedback connections ^75^.

More recently, *in vivo* recordings are beginning to establish how local interneuron circuits modulate learning. During auditory fear conditioning, PV interneurons in the BLA are excited by auditory input (CS) while SOM interneurons are inhibited ^24^. In contrast, PV interneurons are inhibited by footshocks (US) ^24^. Since SOM interneurons in the BLA receive inhibitory input from PV interneurons ^24^, the inhibition of SOM interneurons during CS presentation is proposed to be mediated by PV cells driven by the CS ^24^. Functionally, the CS is thought to support principal neuron dendritic depolarization by disinhibition of SOM interneurons. Finally, input to interneurons can also undergo synaptic plasticity ^82, 84^, and there are clear changes to inhibitory circuitry following fear conditioning ^84, 85^. In summary, inhibition in the BLA is a strong regulator of principal cell activity, and it is clear that inhibitory microcircuits play crucial and cell type-specific roles in fear conditioning ^86^. However, how the activity of these microcircuits establishes CS–US contingency is not clear.

## Fear conditioning: expression

Within the BLA, auditory input is concentrated in the LA ^87^ and behavioral tests found that pretraining lesions of the LA ^88^, but not the BL ^88, 89^, BM ^88^, or entire BA ^90^, blocks auditory fear conditioning. As described above, this learning is thought to result from the plasticity of synapses made by CS input to principal neurons in the LA. Consistent with this result, inhibition of pyramidal neurons in the LA, but not the BA, impairs fear learning ^91^. Following associative learning, fear memory is thought to be stored as a network of excitatory neurons that has been called the engram ^92^. Individual neurons within the network appear to be allocated by their excitability during fear acquisition ^93^, and inactivation of this network of neurons disrupts memory retrieval ^94^. This engram has been associated with pyramidal neurons in the LA ^94^. However, following fear conditioning, a network of neurons driven by the CS has also been identified in the BA ^52^.

The primary target of auditory input is the LA, and the main target for LA pyramidal neurons is the BA ^95, 96^, which in turn send afferents to the CeM ^97^, the main output station of amygdala to hypothalamus and brainstem ^8, 98^. As expected, single unit recordings show selective increase of CS evoked spike firing after auditory fear conditioning in the LA ^10, 46, 99, 100^. In agreement with the anatomy, a significant number of neurons in the BL and BM also acquire CS responsiveness following fear conditioning ^47, 52, 101^. Inactivation of either the BL or the BM individually has little impact on fear expression, but inactivation of the entire BA abolishes fear expression ^47^. These results have led to a model of fear conditioning in which learning requires the LA, fear expression is gated by BLA projections to the central amygdala, and downstream projections from the central amygdala initiate the physiological responses underlying the defensive responses elicited by the CS ^47^.

## Conclusions

In summary, the BLA is a complex structure that plays a central role in cued auditory fear conditioning. During learning, CS and US inputs converge in the LA, and the acquisition of fear memory is driven by contingent CS–US activity that results in an enhanced CS input by a mechanism that requires local inhibitory circuits and activation of NMDA receptors. This enhanced CS activity results in the formation of a memory trace or engram within the BLA. Following fear conditioning, subsequent presentation of the CS retrieves the memory trace by activating a network of neurons in the BA, and the resultant output drives the CeA, initiating the conditioned response. While the evidence for this general model is compelling, the details of the mechanisms that initiate synaptic plasticity, how this plasticity establishes the engram, and the role of local inhibition are not fully understood, and indeed the current literature provides some conflicting observations. With the rapid development of new techniques to interrogate neural function, we have no doubt that these issues are ripe to be settled.
